# From Brown Seaweed to a Sustainable Microbial Feedstock for the Production of Riboflavin

**DOI:** 10.3389/fbioe.2022.863690

**Published:** 2022-04-12

**Authors:** Fernando Pérez-García, Vivien Jessica Klein, Luciana Fernandes Brito, Trygve Brautaset

**Affiliations:** Department of Biotechnology and Food Science, Faculty of Natural Sciences, NTNU, Trondheim, Norway

**Keywords:** seaweed, mannitol, phosphotransferase system, *Corynebacterium glutamicum*, riboflavin, fed-batch fermentation

## Abstract

The increasing global demand for food and energy production encourages the development of new production strategies focused on sustainability. Often, microbial bioprocesses rely on food or feed competitive feedstocks; hence, there is a trending need for green substrates. Here, we have proven the potential of brown seaweed biomass as microbial feedstock on account of its content of mannitol and the glucose polymer laminarin. Our host, *Corynebacterium glutamicum*, was engineered to enable access to mannitol as a carbon source through the heterologous expression of the mannitol-specific phosphotransferase system and the mannitol-1-phosphate-5-dehydrogenase from *Bacillus subtilis.* Overproduction of riboflavin was coupled with mannitol and glucose consumption via constitutive overexpression of the biosynthetic riboflavin operon *ribGCAH* from *C. glutamicum*. Brown seaweed extract and brown seaweed hydrolysate from *Laminaria hyperborea*, containing mannitol and glucose, were used as a carbon source for flask and bioreactor fermentations. In a seaweed-based fed-batch fermentation, the riboflavin final titer, yield, and volumetric productivity values of 1,291.2 mg L^−1^, 66.1 mg g^−1^, and 17.2 mg L^−1^ h^−1^, respectively, were achieved.

## Introduction

Nowadays, there is a continuous and fast increment of the world´s population and, therefore, an increase in the demand for food and energy production, which is commonly associated with environmental, social, and ethical issues. To avoid the dependence on fossil-based fuels and chemicals, the use of new and renewable sources of energy and industry feedstocks is in the spotlight (Barbot et al., 2016). Brown seaweed is a promising feedstock for microbial bioprocesses due to its high growth rates, great biomass production yields, abundance of fermentable carbohydrates, lack of arable land needed, and no freshwater requirement for cultivation. Brown macroalgal biomass, specifically from *Laminaria*, contains up to 60% of fermentable carbohydrates from dry weight (Barbot et al., 2016), including the polysaccharide of glucose, laminarin, and the sugar alcohol mannitol. The bacterium *Corynebacterium glutamicum* appears very suitable to be engineered for the utilization of macroalgae biomass for several reasons. First, *C. glutamicum* is used in amino acid fermentation industry at a million-ton-scale (Becker et al., 2018; Pérez-García and Wendisch, 2018). Moreover, *C. glutamicum* has successfully been engineered for the utilization of non-native carbon sources such as lignocellulosic sugars, glycerol as a by-product of the biodiesel process, or amino sugars from the fishing industry wastes (Wendisch et al., 2016; Becker et al., 2018; Zhang et al., 2020). Finally, *C. glutamicum* lacks a complex carbon catabolite repression system, hence it can consume many substrates in parallel, unlike other biotechnology workhorses like *Escherichia coli* or *Bacillus subtilis* (Görke and Stülke, 2008). Based on previous studies, *C. glutamicum* can utilize mannitol under specific conditions (Laslo et al., 2012). *C. glutamicum* possesses an arabitol/mannitol catabolic operon (Laslo et al., 2012), which comprises three genes (*mtlTDR*). In spite of this, wild-type *C. glutamicum* lacks the ability to utilize mannitol as the sole carbon source. Deletion of the transcriptional repressor gene *mtlR* or the overexpression of the mannitol 2-dehydrogenase (*mtlD*) and the arabitol/mannitol permease (*mtlT*) genes can enable consumption of mannitol. Through this pathway mannitol, is ingested and converted into fructose, which is non-optimal since *C. glutamicum* cannot utilize unphosphorylated fructose. Therefore, fructose has to be released to the medium and then has to be assimilated and phosphorylated through the fructose-specific phosphotransferase system (PTS) (Laslo et al., 2012). On the other hand, the molecule laminarin is a storage linear polysaccharide of glucose made up of β (1→3)-glucan with β (1→6) branches (Becker et al., 2020). While some organisms like *Vibrio* spp. can hydrolysate laminarin releasing glucoses (Alderkamp et al., 2007), *C. glutamicum* lacks the enzymatic machinery to hydrolyze laminarin and, hence, cannot use it as carbon source. However, pretreatment of dissolved laminarin with commercial enzymes containing cellulases, ß-glucosidases, and hemicellulose can release glucose units (Hakvåg et al., 2020). Therefore, it is required to develop tools to enable the utilization of brown seaweed-derived carbohydrates by *C. glutamicum* and, ultimately, the utilization of carbohydrates in seaweed hydrolysate and seaweed extract.

Traditionally used for the million-ton-scale production of amino acids, *C. glutamicum* has become a flexible and efficient production host for various chemicals like non-proteogenic amino acids, diamines, carotenoids, or biofuels (Becker et al., 2018; Pérez-García and Wendisch, 2018). Riboflavin or vitamin B2 is an essential nutrient for higher animals which is involved, for instance, in lipid and energy metabolism and needs to be obtained from the diet (Pinto and Zempleni, 2016; Revuelta et al., 2017). Currently*, B. subtilis* and the filamentous fungus *Ashbya gossypii* are two of the main hosts to produce riboflavin at large scales (Averianova et al., 2020). Such organisms have been extensively engineered to enhance riboflavin production (Schwechheimer et al., 2016; Averianova et al., 2020), although not from alternative and renewable feedstocks. Production of riboflavin by *C. glutamicum* was enabled via overexpression of the sigma factor gene *sigH* (Taniguchi and Wendisch, 2015; Pérez-García et al., 2021). However, the productivity values were not competitive in comparison to those in the current large-scale producers (Averianova et al., 2020).

**FIGURE 1:**
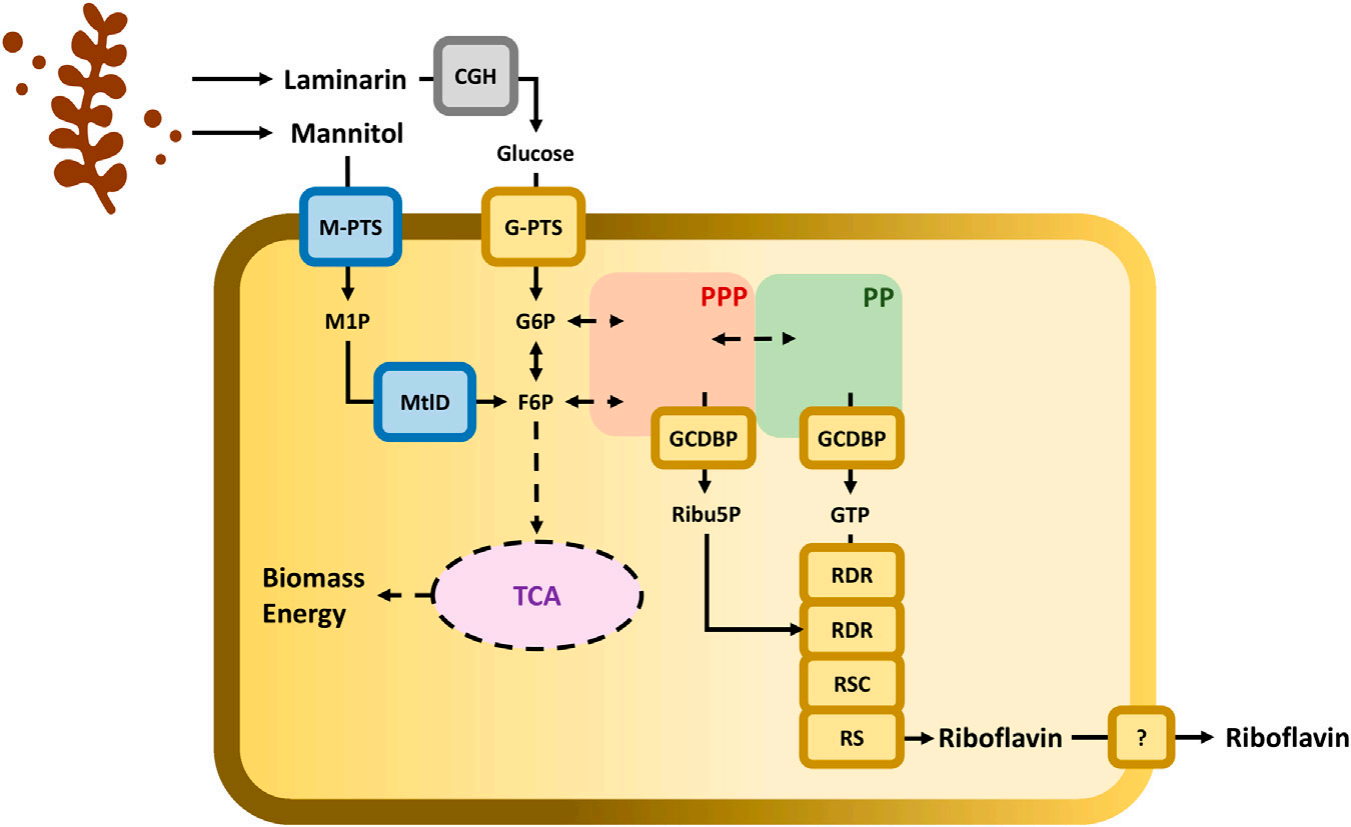
Schematic representation of *C. glutamicum* cell factory with the metabolic connection between the utilization of brown seaweed-derived carbohydrates and the riboflavin biosynthetic pathway. Pathways: The pentose phosphate pathway (PPP) is depicted with a red-shadowed shape; the purine pathway (PP) is depicted with a green-shadowed shape; and tricarboxylic acid (TCA) cycle is depicted with a purple shadowed shape. Enzymes: Endogenous enzymatic steps are depicted in yellow boxes. G-PTS, glucose-specific PTS coded by *ptsG*; GCDBP, GTP cyclohydrolase II/3,4-dihydroxy-2-butanone-4-phosphatesynthase coded by *ribA*; RDR, bifunctional riboflavin-specific deaminase/reductase coded by *ribG*; RSC, riboflavin synthase beta chain coded by *ribH*; and RS, riboflavin synthase by *ribC.* Enzymatic steps implemented in *C. glutamicum* are depicted in blue boxes. M-PTS, mannitol-specific PTS coded by *mtlA or mtlAF;* MtlD, mannitol-1-phosphate-5-dehydrogenase coded by *mtlD*. Pretreatment of laminarin with commercial enzymes containing cellulases, ß-glucosidases, and hemicellulose (CGH) is depicted in grey box. Metabolites: M1P, mannitol 1-phosphate; G6P, glucose 6-phosphase; F6P, fructose 6-phosphate; Ribu5P, ribulose 5-phosphate; and GTP, Guanosine 5-triphosphate.

Hence, we explored the use of mannitol-specific PTSs from mannitol natural microbial consumers in *C. glutamicum*. In combination with a laminarin pretreatment to release glucose, two of the most abundant carbohydrates from brown seaweed were accessible for use by *C. glutamicum* as carbon source. Besides, competitive riboflavin production was achieved and coupled with the synthetic consumption of seaweed substrates in *C. glutamicum* (Figure 1). Finally, scaling-up was achieved in lab-scale bioreactors proving the feasibility of newly constructed strains.

## Materials and Methods

### Bacterial Strains, Plasmids, and Growth Conditions

All plasmids and bacterial strains used in this work are listed in Table 1 and Table 2, respectively. *E. coli* was cultivated in lysogeny broth (LB) or on LB agar plates at 37°C. Pre-cultures of *C. glutamicum* strains were cultivated in brain heart infusion (BHI) medium or BHI agar plates at 30°C. The main cultures of *C. glutamicum* were done in CGXII minimal medium (Eggeling et al., 2005), inoculated to an optical density at 600 nm (OD_600_) of one approx. Optical densities were measured via the spectrophotometer WPA CO 8000 Biowave Cell Density Meter from Biochrom Ltd. All biomass calculations were done by using the correlation factor 1 g L−^1^ biomass = 0.343 OD_600_. When necessary, kanamycin 25 μg ml−^1^, spectinomycin 100 μg ml−^1^, and/or tetracycline 5 μg ml−^1^ were supplemented to the medium. For the induction of genes cloned into the vectors pVWEx6 (Henke et al., 2021) and pEKEx3 (Stansen et al., 2005), the medium was supplemented with different concentrations of isopropyl-β-D-1-thiogalactopyranoside (IPTG), which is specified in Results. All chemicals were purchased from Merck/Sigma. As carbon source, the sugars glucose, mannitol, seaweed extract (SWE), or seaweed hydrolysate (SWH) were added at the concentrations indicated in Results. For the determination of the sugars, organic acids, and riboflavin, samples were withdrawn from the cultures, centrifuged at 16,200 × G and 4°C to remove the cells and stored at −20°C till used. All carbon sources, antibiotics, and biotin were sterilized by filtration, while the rest of the components were sterilized by autoclavation.

**TABLE 1:** List of plasmids used in this work. Kan_R_, kanamycin resistance; Spec^R^, spectinomycin resistance; Tet^R^, tetracycline resistance.

Plasmid name	Description	Source
pVWEx6	Kan^R^, *C. glutamicum*/*E. coli* shuttle plasmid (P_syn_, *lacI*, pHM1519 OriV_Cg_)	Henke et al. (2021)
pEKEx3	Spec^R^, *C. glutamicum*/*E. coli* shuttle plasmid (P_tac_, *lacI*, pBL1 OriV_Cg_)	Stansen et al. (2005)
pECXT-Psyn	Tet^R^, *C. glutamicum*/*E. coli* shuttle plasmid (P_syn_, pGA1 oriV_Cg_)	Henke et al. (2021)
pVWEx6-*mtlDEc*	Kan^R^, pVWEx6 overexpressing *mtlD* from *E. coli MG1655*	This work
pVWEx6-*mtlDBs*	Kan^R^, pVWEx6 overexpressing *mtlD* from *B. subtilis 168*	This work
pVWEx6-*mtlDBm*	Kan^R^, pVWEx6 overexpressing *mtlD* from *B. methanolicus MGA3*	This work
pEKEx3-*mtlAEc*	Spec^R^, pEKEx3 overexpressing *mtlA* from *E. coli MG1655*	This work
pEKEx3-*mtlAFBs*	Spec^R^, pEKEx3 overexpressing *mtlAF* from *B. subtilis 168*	This work
pEKEx3-*mtlAFBm*	Spec^R^, pEKEx3 overexpressing *mtlAF* from *B. methanolicus MGA3*	This work
pECXT-Psyn-*ribGCAH*	Tet^R^, pECXT-Psyn overexpressing the riboflavin biosynthetic operon from *C. glutamicum*	This work

Kan^R^, kanamycin resistance; Spec^R^, spectinomycin resistance; Tet^R^, tetracycline resistance

**TABLE 2:** List of strains used in this work.

Strain Name	Description	Source
C*orynebacterium glutamicum*	Wild-type strain ATCC 13032, auxotrophic for biotin	Abe et al. (1967)
*Escherichia coli*	DH5α Strain:*∆lac*U169 (φ80*lacZ* ∆M15), *sup*E44, *hsd*R17, *rec*A1, *end*A1, *gyr*A96, *thi*-1, *rel*A1	Hanahan (1983)
CgSW1	*C. glutamicum* (pVWEx6)	This work
CgSW2	*C. glutamicum* (pVWEx6-*mtlDEc*)	This work
CgSW3	*C. glutamicum* (pVWEx6-*mtlDBs*)	This work
CgSW4	*C. glutamicum* (pVWEx6*-mtlDBm*)	This work
CgSW5	*C. glutamicum* (pEKEx3)	This work
CgSW6	*C. glutamicum* (pEKEx3-*mtlAEc*)	This work
CgSW7	*C. glutamicum* (pEKEx3-*mtlAFBs*)	This work
CgSW8	*C. glutamicum* (pEKEx3-*mtlAFBm*)	This work
CgSW9	*C. glutamicum* (pVWEx6) (pEKEx3)	This work
CgSW10	*C. glutamicum* (pVWEx6-*mtlDEc*) (pEKEx3-*mtlAEc*)	This work
CgSW11	*C. glutamicum* (pVWEx6-*mtlDBs*) (pEKEx3-*mtlAFBs*)	This work
CgSW12	*C. glutamicum* (pVWEx6-*mtlDBm*) (pEKEx3-*mtlAFBm*)	This work
CgRibo1	*C. glutamicum* (pECXT-Psyn)	This work
CgRibo2	*C. glutamicum* (pECXT-Psyn-*ribGCAH*)	This work
CgRibo3	CgSW9(pECXT-Psyn)	This work
CgRibo4	CgSW9(pECXT-Psyn-*ribGCAH*)	This work

### Molecular Genetics Techniques and Strain Construction

Standard molecular genetics techniques were performed as described elsewhere (Green and Sambrook, 2012). *E. coli* cells were transformed by heat shock (Green and Sambrook, 2012) and *C. glutamicum* cells were transformed by electroporation with a single pulse at 2.5 kV, 200 Ω, and 25 µF (Eggeling et al., 2005). CloneAmp™ HiFi PCR Premix Protocol (Takara Bio Inc.) was used for cloning PCR. GoTaq® DNA polymerase Protocol (Promega) was used for colony PCR. The genes *mtlDEc* and *mtlAEc* were amplified from genomic DNA from *E. coli* MG1655 with the primers mtlD-Ec-Fw, mtlD-Ec-Rv, mtlA-Ec-Fw, and mtlA-Ec-Rv. The genes *mtlDBs* and *mtlAFBs* were amplified from genomic DNA from *B. subtilis* str. 168 with the primers mtlD-Bs-Fw, mtlD-Bs-Rv, mtlAF-Bs-Fw, and mtlAF-Bs-Rv. The genes *mtlDBm*, *mtlABm*, and *mtlFBm* were amplified from genomic DNA from *Bacillus methanolicus* MGA3 with the primers mtlD-Bm-Fw, mtlD-Bm-Rv, mtlA-Bs-Fw, mtlA-Bs-Rv, mtlF-Bs-Fw, and mtlF-Bs-Rv. The whole riboflavin biosynthetic operon (*ribGCAH*) from *C. glutamicum* was amplified from genomic DNA from *C. glutamicum* ATCC13021 with the primers RibCg-Fw and RibCg-Rv. Colony PCRs for the pEKEx3-based cloning were carried out with the primers X3Fw and X3Rv. Colony PCRs for the pVWEx6-based cloning were carried out with the primers X6Fw and X6Rv. Colony PCRs for the pECXT-Psyn-based cloning were carried out with the primers SYFw and SYRv. All primers were purchased from Sigma, and all primer sequences are listed in Table 3. The plasmids pVWEx6 (Henke et al., 2021), pEKEx3 (Stansen et al., 2005), and pECXT-Psyn (Henke et al., 2021) were restricted with BamHI (New England Biolabs) and were combined with the amplified PCR products in Gibson Assembly (Gibson et al., 2009) to construct the plasmids listed in Table 1.

**TABLE 3:** List of primers used in this work.

Primer Name	Sequence (5' - 3′)
X3Fw	CATCATAACGGTTCTGGC
X3Rv	ATCTTCTCTCATCCGCCA
X6Fw	ATG​CCG​CTT​CGC​CTT​CGT​TG
X6Rv	CGA​CGG​CCA​GTG​AAT​TCG​AG
SYFw	ACG​CAT​CTG​TGC​GGT​ATT​TC
SYRv	TAC​GGC​GTT​TCA​CTT​CTG​AG
mtlD-Ec-Fw	**GGA​ATT​CGA​GCT​CGG​TAC​CCG​GG** GAA​AGG​AGG​CCC​TTC​AGATG​AAA​GCA​TTA​CAT​TTT​GGC
mtlD-Ec-Rv	**CTG​CAG​GTC​GAC​TCT​AGA​GGA​TC**TTA​TTG​CAT​TGC​TTT​ATA​AGC​G
mtlD-Bs-Fw	**GGA​ATT​CGA​GCT​CGG​TAC​CCG​GG** GAA​AGG​AGG​CCC​TTC​AGATG​ATC​GCC​TTA​CAT​TTC​GG
mtlD-Bs-Rv	**CTG​CAG​GTC​GAC​TCT​AGA​GGA​TC**TTA​TTG​ATT​AAG​TTT​CTT​TAA​AAT​G
mtlD-Bm-Fw	**GGA​ATT​CGA​GCT​CGG​TAC​CCG​GG** GAA​AGG​AGG​CCC​TTC​AGATG​CTA​GCT​GTG​CAT​TTC​GG
mtlD-Bm-Rv	**CTG​CAG​GTC​GAC​TCT​AGA​GGA​TC**TTA​TAA​TTT​CCC​GCC​CTT​AAA​TG
mtlA-Ec-Fw	**CAT​GCC​TGC​AGG​TCG​ACT​CTA​GAG** GAA​AGG​AGG​CCC​TTC​AGATG​TCA​TCC​GAT​ATT​AAG​ATC
mtlA-Ec-Rv	**CGA​GCT​CGG​TAC​CCG​GGG​ATC**TTA​CTT​ACG​ACC​TGC​CAG​CAG
mtlAF-Bs-Fw	**CAT​GCC​TGC​AGG​TCG​ACT​CTA​GAG** GAA​AGG​AGG​CCC​TTC​AGATG​CAG​CAG​CAA​GAA​CAG​CAG
mtlAF-Bs-Rv	**CGA​GCT​CGG​TAC​CCG​GGG​ATC**TCA​GTT​CAC​CTC​GTT​GAA​AAT​GG
mtlAF-Bm-Fw	**CAT​GCC​TGC​AGG​TCG​ACT​CTA​GAG** GAA​AGG​AGG​CCC​TTC​AGATG​ACG​AAT​ACG​AAT​CAG​TC
mtlA-Bm-Rv	**CTT​TCG​GGG​CGT​TCG​AA**AGC​CTC​GCC​TCC​AAA​ACG
mtlF-Bm-Fw	**GAG​GCT** *TTC​GAA​CGC​CCC* GAA​AGG​AGG​CCC​TTC​AGATG​GCT​TTA​CCA​ATC​TTA​TC
mtlAF-Bm-Rv	**CGA​GCT​CGG​TAC​CCG​GGG​ATC**TTA​GTT​CAC​TCC​TTC​AAA​G
RibCg-Fw	**CAT​GGA​ATT​CGA​GCT​CGG​TAC​CCG​GG** GAA​AGG​AGG​CCC​TTC​AGATG​GAT​GTT​GCG​CAC​GCG
RibCg-Rv	**GCC​TGC​AGG​TCG​ACT​CTA​GAG​GAT​C**CTA​ACC​CTC​AGT​TGC​ACG

Bold letters: overlapping region. Underlined letters: ribosomal biding site plus spacer. Italic letters: link sequence.

### Preparation of Seaweed Substrates

As study material for this work, brown seaweed biomass and brown SWH from *Laminaria hyperborea* was kindly provided by the company Alginor in Norway. For the preparation of SWE, frozen and milled *Laminaria hyperborea* leaf biomass was thawed overnight at 4°C. Hot water (70°C approx.) was added to the biomass with the proportion of 1:1 (grams biomass: grams water). The pH was then adjusted to 3.5 by the addition of sulfuric acid. Next, the mixture was incubated at 70°C for 2 to 3 h. When still hot, the mixture was centrifuged at 47,800 × G for 20 min. The supernatant was collected, and the biomass was discarded. For the enzymatic digestion of laminarin, the pH was adjusted to 5.0, and 1 ml of the enzyme blend Cellic CTect2 (Merck/Sigma) was added per 100 ml of supernatant, followed by incubation at 50°C and the mixture was shaken at 200 rpm for 24 h. The pH of the enzyme-treated supernatant was adjusted to 7.0 and the substrate was sterile-filtered before use. The *L. hyperborea* hydrolysate (SWH) provided by the company Alginor was also treated with the enzyme blend Cellic CTect2 as described previously (Hakvåg et al., 2020).

### Analytical Procedures

For the quantification of extracellular sugars, furans (furfural and 5-hydroxymethylfurfural), and riboflavin, a high-pressure liquid chromatography (HPLC) system was used (Waters Alliance e2695 Separations Module). Supernatants of culture broth were diluted in a proportion of 1:10 before analyzing and storing at −20°C. The quantification of sugars and furans was performed using a 300 mm × 7.7 mm Hi-Plex H column (Agilent Technologies) prewarmed at 45°C and detected by a refractive index detector (2414 RI Detector, Waters). Sulfuric acid of 5 mM was used as mobile phase at a flow rate of 0.6 ml min^−1^ . The quantification of riboflavin was performed using a 75 mm × 4.6 mm Symmetry C18 Column 3.5 µm column (Waters) prewarmed at 25°C, and detected by a fluorescence detector (2475 FLR Detector, Waters) with the excitation and emission wavelengths of 370 and 520 nm, respectively. Ammonium acetate of 5 mM with a pH of 6.0 and methanol (3:1) was used as mobile phase at a flow rate of 0.8 ml min^−1^ . Sample preparation for riboflavin quantification was carried out as described in a previous study (Petteys and Frank, 2011).

### Bioreactor Conditions

Baffled glass autoclavable bioreactors with a total volume of 2 L (working volume of 1.7 L) were used (Applikon Biotechnology). Two four-bladed Rushton impellers with outer diameters of 45 mm were placed in the stirrer axis with a distance from the bottom of the reactor of 6 and 12 cm. The relative dissolved oxygen saturation (rDOS) was automatically controlled and maintained via the stirring rpm. The rDOS levels were monitored with 12 mm polarographic DO_2_ sensors (AppliSens). A pH of 7.0 was established and controlled by the automatic addition of phosphoric acid (10 % (w/w)) and potassium hydroxide (4 M). The pH values were monitored with 12 mm pH sensors (AppliSens). Antifoam 204 (Sigma) was added manually when needed. The cultivation temperature was maintained at a constant temperature of 30°C using a heating jacket via a bioreactor wall. A constant aeration rate of 0.75 vvm of air was applied from the bottom of the bioreactor with a L-type sparger.

The initial working volume of 0.7 L was inoculated to an OD_600_ of five from the overnight shake flask pre-culture in a complex medium 2 YT (16 g of tryptone 10 g yeast extract and 5 g of NaCl per liter). Samples were collected manually using Super Safe Samplers (Infors HT) and used for OD_600_ measurements and for the preparation of HPLC samples. Per liter of batch medium, it contained: 10 g (NH_4_)_2_SO_4_, 5 g Urea, 0.5 g KH_2_PO_4_, 0.5 g K_2_HPO_4_, 0.01325 g CaCl_2_ x 2H_2_O, 0.25 g MgSO_4_ x 7H_2_O, 0.2 mg biotin, and the trace elements 1 mg FeSO_4_ x 7H_2_O, 1 mg MnSO_4_ x H_2_O, 0.1 mg ZnSO_4_ x 7H_2_O, 0.02 mg CuSO_4_, and 0.002 mg NiCl_2_ x 6H_2_O. The batch medium contained 80% (v/v) of SWE or SWH. The feed medium contained 100% (v/v) of SWE or SWH. Linear feeding rates of 2.5 ml min^−1^ were manually initiated when the rDOS values increased from 30% to 65% for the first time. The processes were completed, which was indicated by the increase of the rDOS values from 30 to 65 % during the feeding phase.

## Results

### Establishing Mannitol Consumption in *C. glutamicum* via Mannitol-Specific PTS

**FIGURE 2:**
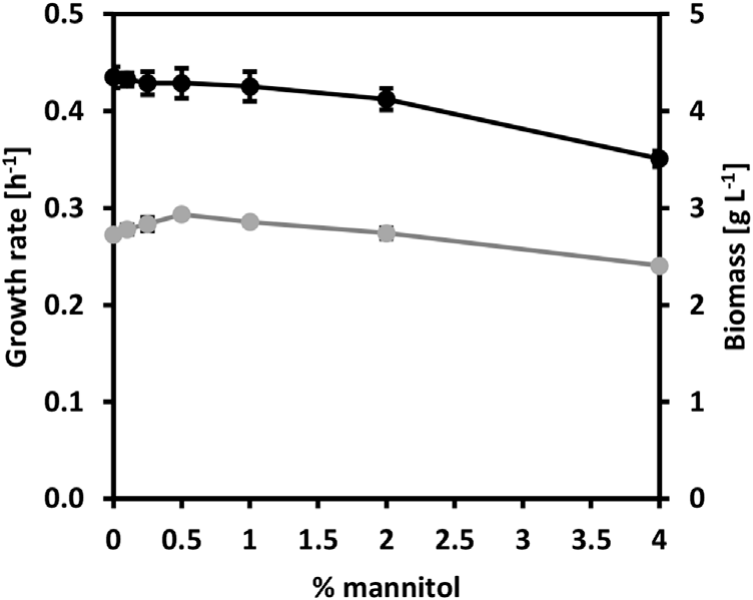
Growth behavior of strain CgSW9 in the presence of mannitol. Glucose-based minimal medium was supplemented with gradually increasing concentrations of mannitol. Growth rate values are depicted in grey. Biomass values are depicted in black. Means and standard deviations of triplicates are shown.

**FIGURE 3:**
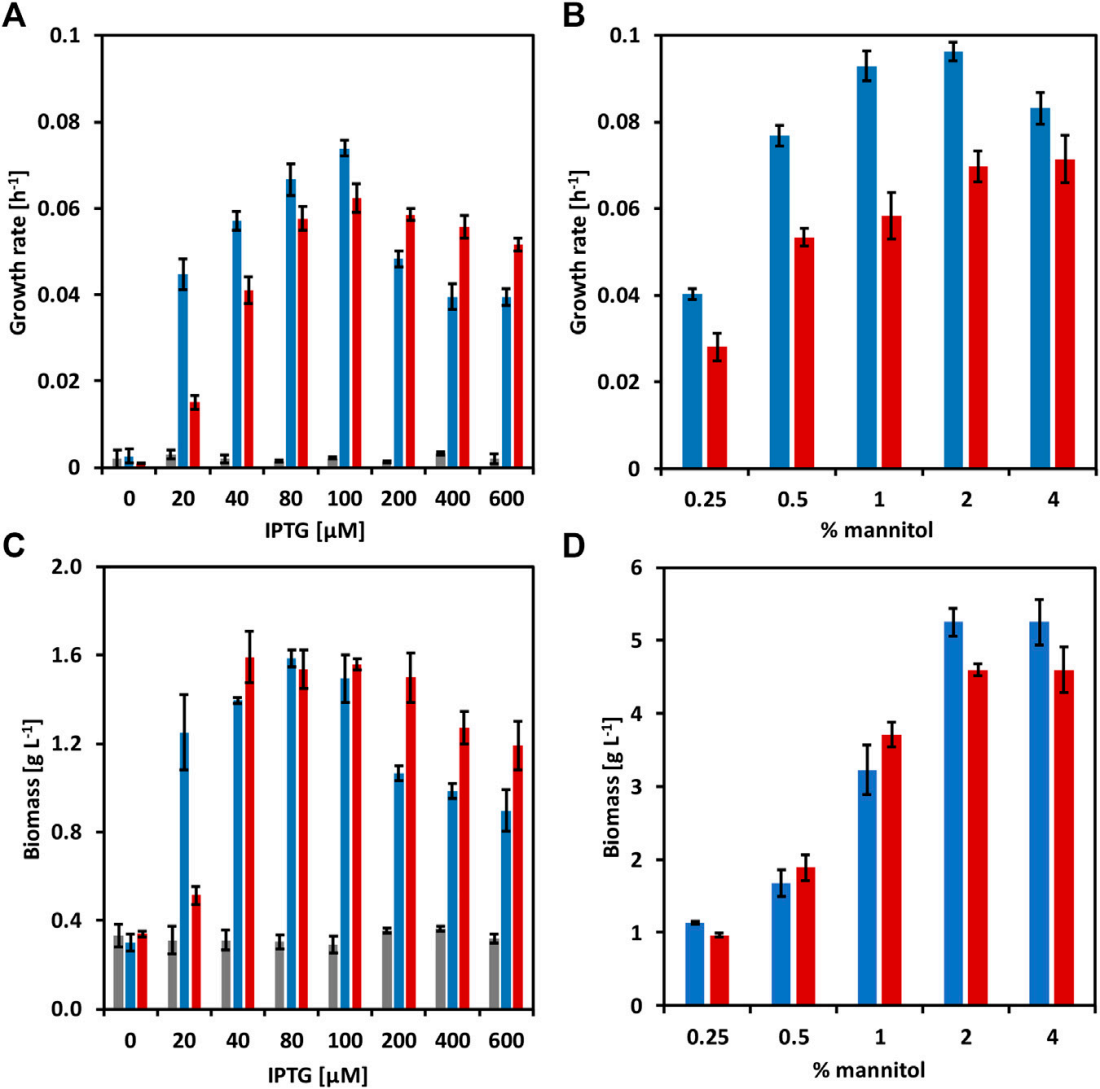
Growth behavior of the strains CgSW10, CgSW11, and CgSW12 with mannitol as sole carbon source. **(A)** Growth rates of CgSW10 (grey columns), CgSW11 (blue columns), and CgSW12 (red columns) strains grown in 0.5% mannitol minimal medium supplemented with different concentrations of IPTG. **(B)** Growth rates of CgSW11 and CgSW12 strains grown in minimal medium supplemented with different concentrations mannitol and 100 µM of IPTG. **(C)** Final biomass of CgSW10, CgSW11, and CgSW12 strains grown in 0.5% mannitol minimal medium supplemented with different concentrations of IPTG. **(D)** Final biomass of CgSW11 and CgSW12 strains grown in minimal medium supplemented with 100 µM of IPTG and different concentrations of mannitol. Means and standard deviations of triplicates are shown.

Mannitol, a type of sugar alcohol, is one of the most abundant energy and carbon storage molecules in nature, which is also present in brown seaweed biomass (Song and Vieille, 2009; Barbot et al., 2016). However, mannitol is not a natural substrate for *C. glutamicum.* When growing *C. glutamicum* (pVWEx6) (pEKEx3) (named CgSW9) in minimal medium supplemented with 1% of glucose along with gradually increasing concentrations of mannitol (Figure 2), we observed that the growth rates of *C. glutamicum* slightly increased with mannitol concentrations from 0 to 1%, having a negative impact in growth rate and biomass formation at 4% of mannitol. HPLC data indicated that only glucose was depleted. *C. glutamicum* can utilize mannitol under certain conditions (Laslo et al., 2012). However, the utilization of mannitol by *C. glutamicum* via mannitol-specific PTS remains unexplored. *C. glutamicum* lacks a mannitol-specific PTS (Laslo et al., 2012), hence we assessed mannitol-specific PTS from different microorganisms that were able to consume mannitol through a PTS. The genes for the mannitol-1-phosphate-5-dehydrogenase (*mtlD*) and the mannitol-specific PTS genes *mtlAF* from *E. coli*, *B. subtilis*, and *B. methanolicus* were tested and evaluated in *C. glutamicum* wild-type. While MtlD is a cytosolic protein that converts mannitol-1-phosphate in fructose 1-phosphate, MtlAF are membrane proteins that participate in the uptake of mannitol and its phosphorylation to mannitol 1-phosphate. The *mtlD* genes were cloned into the IPTG-inducible expression vector pVWEx6 [Henke et al., 2021). Transformation of *C. glutamicum* with the *mtlD* carrying vectors and the empty vector yielded the strains CgSW1 (*C. glutamicum* (pVWEx6)], CgSW2 [*C. glutamicum* (pVWEx6-*mtlDEc*)], CgSW3 [*C. glutamicum* (pVWEx6-*mtlDBs*)], and CgSW4 [*C. glutamicum* (pVWEx6-*mtlDBm*)] (Table 2). On the other hand, the *mtlAF* genes were cloned into the IPTG-inducible expression vector pEKEx3 (Stansen et al., 2005). Transformation of *C. glutamicum* with the *mtlAF* carrying vectors and the empty vector yielded the strains CgSW5 [*C. glutamicum* (pEKEx3)], CgSW6 [*C. glutamicum* (pEKEx3-*mtlAEc*)], CgSW7 [*C. glutamicum* (pEKEx3-*mtlAFBs*)], and CgSW8 [*C. glutamicum* (pEKEx3-*mtlAFBm*)] (Table 2). Finally, the combination of the pVWEx6- and pEKEx3-derived vectors in *C. glutamicum* yielded the strains CgSW9 [*C. glutamicum* (pVWEx6) (pEKEx3)], CgSW10 [*C. glutamicum* (pVWEx6-*mtlDEc*) (pEKEx3-*mtlAEc*)], CgSW11 [*C. glutamicum* (pVWEx6-*mtlDBs*) (pEKEx3-*mtlAFBs*)], and CgSW12 [*C. glutamicum* (pVWEx6-*mtlDBm*) (pEKEx3-*mtlAFBm*)] (Table 2). All the strains were tested under fermentation conditions for 48 h with minimal medium inoculated from the BHI pre-cultures to an OD_600_ of around one and supplemented with 0.5% of mannitol as sole carbon source and different IPTG concentrations (ranging from 0 to 600 µM). However, the single vector strains did not grow and, hence, native mannitol-1-phosphate-5-dehydrogenase and mannitol-specific PTS activities were not detected under the conditions tested (data not shown). Instead, the double vector strains carrying the genes from *B. subtilis* and *B. methanolicus* showed growth (Figures 3A,C). Under the conditions assayed, the growth rate of CgSW11 and CgSW12 peaked at 0.07 ± 0.00 h^−1^ and 0.06 ± 0.00 h^−1^ when using 100 µM of IPTG. Higher IPTG concentrations showed negative impact in the growth rates of CgSW11 and CgSW12 (Figure 3A). The control strain CgSW10 and the strain overexpressing the PTS genes from *E. coli* did not grow. As for the biomass formed, CgSW11 and CgSW12 were able to reach concentrations of about 1.6 g L^−1^. A negative impact in the biomass formation was observed from 200 µM of IPTG for CgSW11 and from 400 µM of IPTG for CgSW12 (Figure 3C). The genes from *B. subtilis* are more harmful than those from *B. methanolicus* for *C. glutamicum* since high induction via IPTG causes an important drop in the growth rate and biomass formation (Figures 3A,C).

CgSW11 and CgSW12 strains were further tested in minimal medium supplemented with increasing concentrations of mannitol as sole carbon source and 100 µM of IPTG (Figures 3B,D). Under those conditions, CgSW11 grew faster at 1 and 2 % of mannitol with growth rates of 0.09 ± 0.00 h^−1^and 0.10 ± 0.00 h^−1^, respectively. CgSW12 grew faster at 2 and 4% of mannitol with growth rates of 0.07 ± 0.00 h^−1^ and 0.07 ± 0.01 h^−1^, respectively (Figure 3B). When using 2 and 4% of mannitol, CgSW11 reached a biomass of 5.2 g L^−1^, while under the same mannitol concentrations, CgSW12 reached a slightly lower biomass of 4.6 g L^−1^ (Figure 3D). The strain CgSW11 seems to perform better at lower mannitol concentrations, although it grew faster than CgSW12 under all mannitol concentrations tested here (Figures 3B,D).

### Establishing Brown Seaweed as Microbial Feedstock for *C. glutamicum*

Apart from mannitol, brown seaweed also contains large reserves of laminarin (Barbot et al., 2016). *C. glutamicum* lacks the laminarin-degrading enzyme systems and hence, cannot utilize laminarin as carbon source. Therefore, in this work, laminarin was enzymatically predigested to release glucose.

The strains CgSW9, CgSW11, and CgSW12 were tested under fermentation for 48 h with minimal medium with glucose, mannitol, and glucose along with mannitol as sole carbon source. Data for the growth rate, biomass formation, and biomass yield were collected and depicted in Table 4. The control strain CgSW9 only grew when glucose was supplemented in the medium, independently of the presence of mannitol. The growth rate of CgSW9 with only glucose was 0.25 ± 0.02 h^−1^ and with glucose plus mannitol was 0.26 ± 0.00 h^−1^. The strains CgSW11 and CgSW12 could grow with mannitol, glucose, and with the combination of both sugars (Table 4). The growth rate, biomass, and biomass yield values for both strains with glucose as sole carbon source were similar to those of the control strain CgSW9 (Table 4). As shown previously (Figures 3A,B), CgSW11 could grow faster on mannitol than CgSW12 (Table 4). The main differences observed were from the cultures with the combined carbon sources glucose and mannitol. CgSW11 grew slightly at a slower rate (0.23 ± 0.01 h^−1^) combined with both sugars together than only with glucose (0.26 ± 0.00 h^−1^). However, CgSW12 showed a more dramatic drop in the growth rate when growing on mannitol and glucose (0.18 ± 0.01 h^−1^) as compared to its growth only with glucose (0.25 ± 0.01 h^−1^) (Table 4). The biomass and yield values from the CgSW12 as compared to those from CgSW9 and CgSW11 indicated the possibility that not all sugars were fully depleted. This was confirmed via HPLC analysis, since more than 9 g L^−1^ of mannitol were detected in the supernatants of CgSW12 when the growth was completed. The fact that the proteins from *B. methanolicus* showed some sort of competition for glucose and mannitol made the strain CgSW12 not suitable for the purpose of this study. Therefore, the CgSW11 strain was chosen for further steps.

**TABLE 4:** Growth values of the strains CgSW9, SgSW11, and CgSW12 when using mannitol, glucose, or mannitol plus glucose as sole carbon sources.

Strain	% Mannitol	% Glucose	Growth rate	Biomass	Yield
			(h^−1^)	(g L^−1^)	(g g^−1^)
CgSW9	1	0	0.00 ± 0.00	0.0 ± 0.0	0.00 ± 0.00
	0	1	0.25 ± 0.02	4.0 ± 0.2	0.40 ± 0.02
	1	1	0.26 ± 0.00	3.9 ± 0.3	0.20 ± 0.03
CgSW11	1	0	0.09 ± 0.01	3.5 ± 0.1	0.35 ± 0.01
	0	1	0.26 ± 0.00	4.0 ± 0.4	0.40 ± 0.04
	1	1	0.23 ± 0.01	6.8 ± 0.5	0.34 ± 0.05
CgSW12	1	0	0.06 ± 0.01	3.0 ± 0.2	0.30 ± 0.02
	0	1	0.25 ± 0.01	4.3 ± 0.2	0.43 ± 0.02
	1	1	0.18 ± 0.01	4.1 ± 0.4	0.21 ± 0.04

For laminarin hydrolysis, the enzyme blend Cellic CTect2, which contains cellulases, ß-glucosidases, and hemicellulose was applied to the SWE and the SWH. The content of mannitol and glucose in the SWE and SWH was quantified via HPLC before and after the laminarin hydrolysis treatment, and the results can be seen in Table 5.

**TABLE 5:** Mannitol and glucose concentrations in the seaweed extract (SWE) and seaweed hydrolysate (SWH) used in this work.

	Mannitol (g L^−1^)	Glucose (g L^−1^)
Seaweed extract (SWE)*	4.73 ± 0.25	-
Seaweed extract (SWE)	4.55 ± 0.15	12.53 ± 0.33
Seaweed hydrolysate (SWH)*	7.32 ± 0.15	-
Seaweed hydrolysate (SWH)	7.02 ± 0.42	6.22 ± 0.47

*Data obtained before the hydrolysis of laminarin.

**FIGURE 4:**
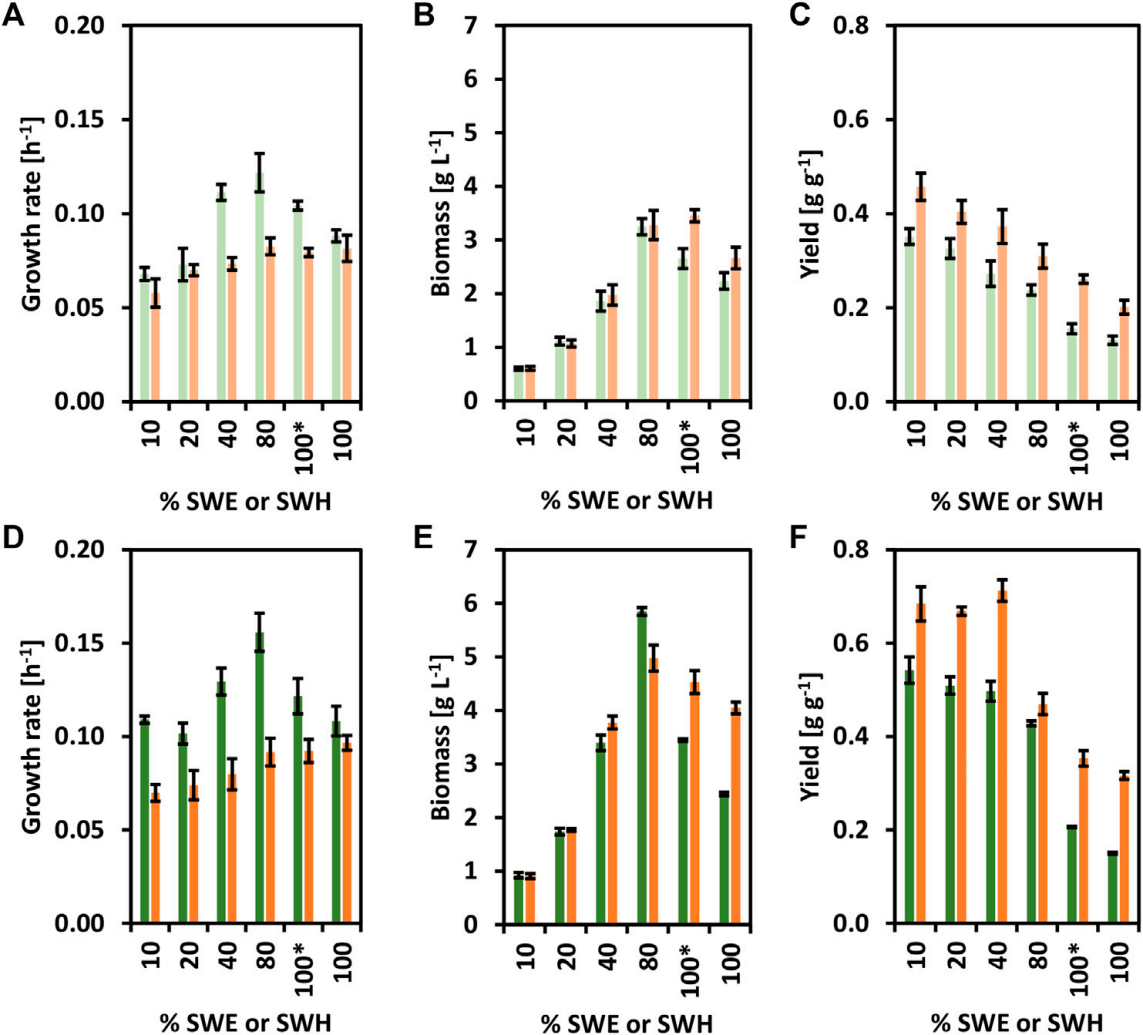
Growth of the strains CgSW9 and CgSW11 in brown seaweed-based substrates. Concentrations of SWE or SWH that ranged from 10 to 100% were used as carbon source. The condition 100* means 100% seaweed-based substrate supplemented with trace elements and biotin. **(A)** Growth rates (h^−1^) of CgSW9. **(B)** Final biomasses formed (g L^−1^) of CgSW9. **(C)** Biomass yields (g g^−1^) of CgSW9. **(D)** Growth rates (h^−1^) of CgSW11. **(E)** Final biomasses formed (g L^−1^) of CgSW11. **(F)** Biomass yields (g g^−1^) of CgSW11. Values from CgSW9 grown on SWE are depicted with light green columns. Values from CgSW9 grown on SWH are depicted with light orange columns. Values from CgSW11 grown on SWE are depicted with dark green columns. Values from CgSW11 grown on SWH are depicted with dark orange columns. Means and standard deviations of triplicates are shown.

Next, the strains CgSW9 and CgSW11 were grown on minimal medium supplemented with different % of SWE or SWH as carbon source (Figure 4). Both strains were able to grow using SWH and SWE. While CgSW9 and CgSW11 grew faster with SWE than with SWH (Figures 4A,D), the biomass yields were higher with SWH than with SWE (Figures 4B,E). Hence, even though SWH may contain additional substrates apart from glucose and mannitol, SWE seems less harmful for the cells. When measuring furans as growth inhibitors via HPLC, 0.42 ± 0.04 mM of furfural was detected only in SWH. A growth rate of 0.16 ± 0.01 h^−1^ and a biomass of 5.8 0.1 g L^−1^ were achieved when using the strain CgSW11 and 80% of SWE as carbon source; the highest growth rate and biomass values were attained under the conditions tested here (Figures 4D,E). In addition, CgSW9 and CgSW11 were also tested with 100 % of SWE or SWH, with and without the addition of trace elements and biotin. However, the growth rate and biomass values were, in general, lower in comparison with the values obtained from the cultivations with SWE or SWH combined with minimal medium CGXII, especially with SWE and when no trace elements and biotin were supplemented (Figures 4A–E). Hence, SWH seems to cope with (Figures 4A–E) the lack of micronutrients needed for the cultivation of *C. glutamicum* strains to a certain extent.

Concluding this block, CgSW11 proved to be a suitable strain for the consumption of brown seaweed-based substrates, better growth values can be achieved via a combination of minimal medium together with SWE or SWH. It is also noteworthy that SWH is a richer medium than SWE, but more harmful for *C. glutamicum*.

### Efficient Riboflavin Production From Glucose and Seaweed-Based Substrates With *C. glutamicum*

**FIGURE 5:**
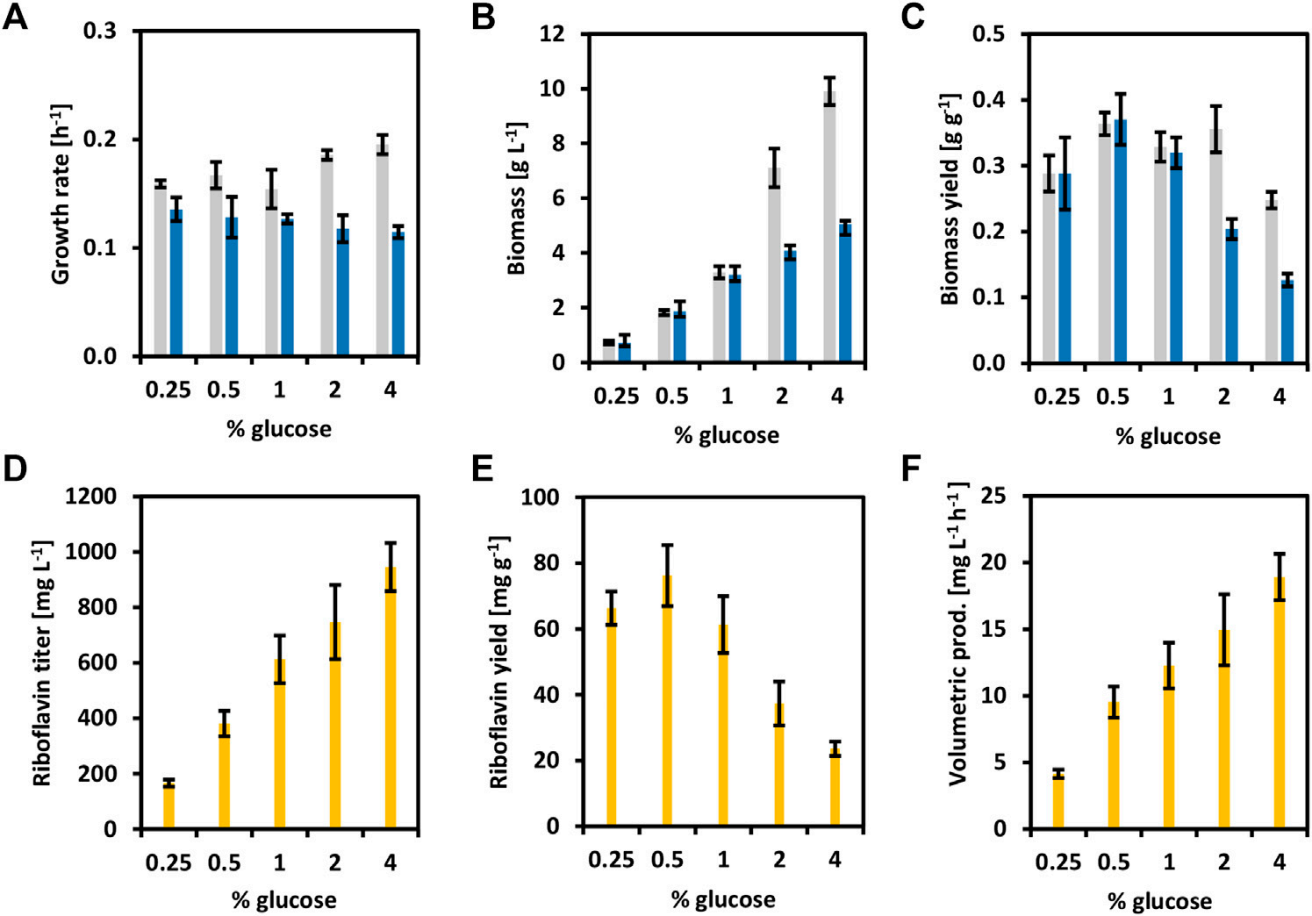
Growth and riboflavin production from glucose of the strains CgRibo1 and CgRibo2. Concentrations of glucose ranged from 0.25 to 4 g L^−1^ were tested. Growth rates (h^−1^) **(A)**, final biomasses formed (g L^−1^) **(B)**, and biomass yields (g g^−1^) **(C)** of CgRibo1 (grey) and CgRibo2 (blue) are depicted. Production values of final riboflavin titers (mg L^−1^) **(D)**, riboflavin yields (mg g^−1^) **(E)**, and riboflavin volumetric productivities (mg L^−1^ h^−1^) **(F)** of CgRibo2 are depicted with yellow columns. Means and standard deviations of triplicates are shown.

**FIGURE 6:**
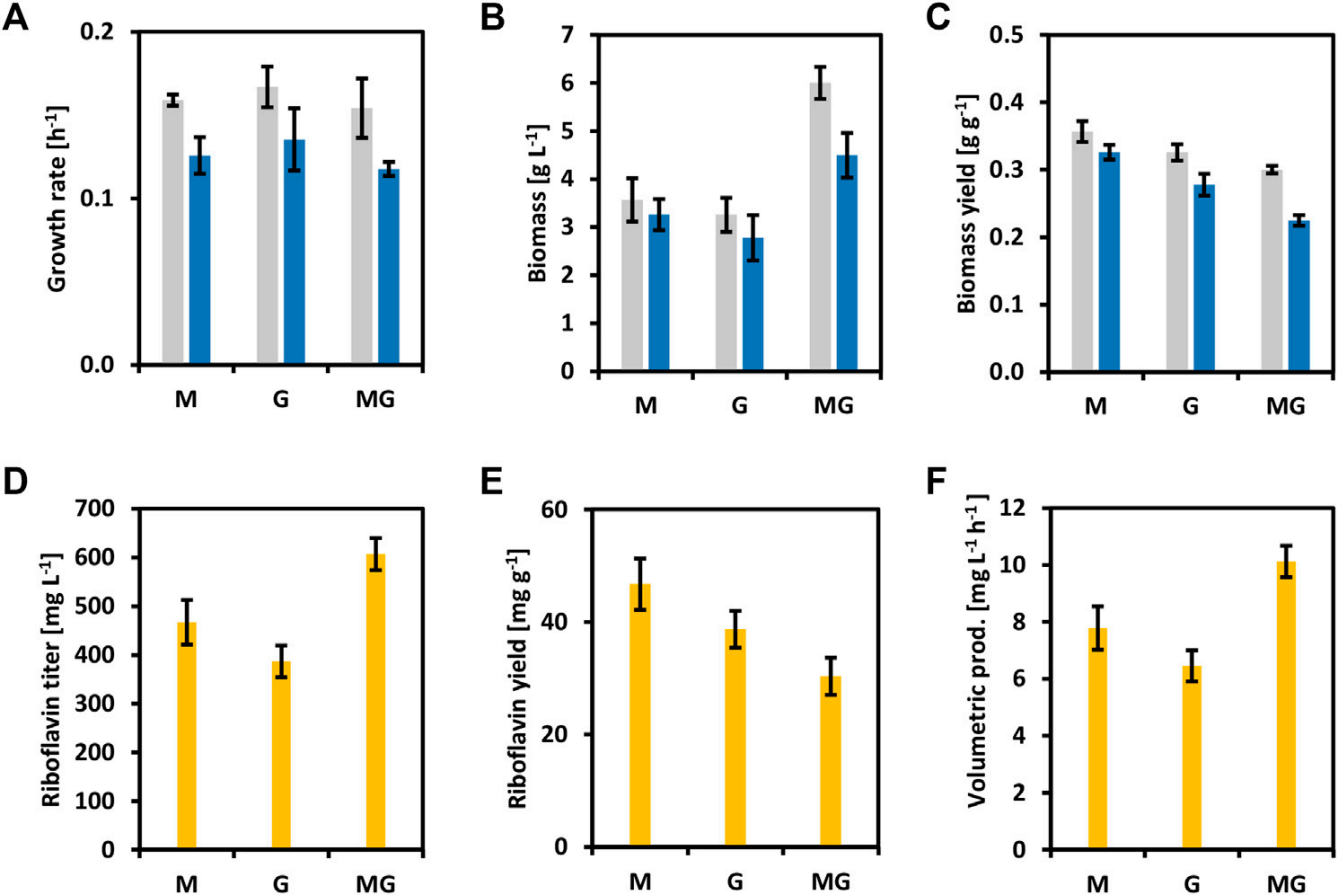
Growth and riboflavin production from brown seaweed sugars of the strains CgRibo3 and CgRibo4. 1% of glucose, 1% of mannitol, and 1% of glucose and 1% of mannitol were assayed. **(A)** Growth rates (h^−1^) of CgRibo3 and CgRibo4. **(B)** Final biomasses of formed (g L^−1^) CgRibo3 and CgRibo4. **(C)** Biomass yields (g g^−1^) of CgRibo3 and CgRibo4. **(D)** Final riboflavin titers (mg L^−1^) of CgRibo4. **(E)** Riboflavin yields (mg g^−1^) of CgRibo4. **(F)** Riboflavin volumetric productivities (mg L^−1^ h^−1^) of CgRibo4. Growth values of CgRibo3 are depicted with grey columns. Growth values of CgRibo4 are depicted with blue columns. Production values of CgRibo4 are depicted with yellow columns. Means and standard deviations of triplicates are shown.

To reinforce the concept of circular bioeconomy, a newly added value chain from brown seaweed was created here. Riboflavin and its active forms have been extensively used in the food, feed, and pharmaceutical industries (Liu et al., 2020). To overproduce riboflavin with *C. glutamicum*, the whole riboflavin biosynthetic operon (*ribGCAH*, Figure 1) from *C. glutamicum* was cloned into the vector pECXT-Psyn under the control of the constitutive promoter P_syn_ (Henke et al., 2021). The vectors pECXT-Psyn and pECXT-Psyn-*ribGCAH* were transferred to *C. glutamicum* wild-type yielding the strains CgRibo1 and CgRibo2 (Table 2). Growth and production of CgRibo1 and CgRibo2 were tested in minimal medium supplemented with different concentrations of glucose as sole carbon source (Figure 5). The highest riboflavin titer achieved by the strain CgRibo2 was 944.6 ± 87.1 mg L^−1^ using 4% of glucose and within 50 h, meaning a volumetric productivity of 18.9 ± 1.7 mg L^−1^ h^−1^ (Figures 5D–F). However, biomass and riboflavin yields were affected when supplementing 2 and 4% of glucose (Figures 5C–E). Growth rates of CgRibo2 were also impaired as compared to those from CgRibo1 when the strain was fed with high glucose concentrations (Figure 5A). Nevertheless, the vector pECXT-Psyn-*ribGCAH* proved to be an excellent tool to establish riboflavin production in *C. glutamicum* strains. Therefore, the vector pECXT-Psyn was transferred to the strain CgSW9 and the vector pECXT-Psyn-*ribGCAH* was transferred to the strain CgSW11, yielding the strains CgRibo3 and CgRibo4, respectively (Table 2). Afterward, growth and production of CgRibo3 and CgRibo4 were tested in minimal medium supplemented with 1% mannitol, 1% glucose, or 1% mannitol and 1% glucose (Figure 6). In general, CgRibo4 grew at a slower rate and reached lower biomass yields than CgRibo3 under all conditions tested here. Particularly, when using glucose and mannitol as carbon source, the biomass formation was drastically lower for CgRibo4 than for CgRibo3 (Figures 6A–C). The riboflavin titer of CgRibo4 with 1% glucose as sole carbon source was 387.1 ± 32.7 mg L^−1^, which is 41.6% lower titer as compared with the riboflavin titer achieved by CgRibo2 with 1% glucose as sole carbon source. Hence, the riboflavin production is impaired by the metabolic modifications implemented in CgRibo4. When using 1% mannitol as sole carbon source, CgRibo4 produced 467.3 ± 45.7 mg L^−1^ of riboflavin, which is17.2% higher titer than the one obtained from 1% glucose (Figure 6D). Finally, when using 1% glucose together with 1% mannitol as carbon source, the CgRibo4 strain produced 607.3 ± 33.1 mg L^−1^ of riboflavin, implying a riboflavin yield of 30.4 ± 3.3 mg g^−1^ (Figures 6D–F).

**FIGURE 7:**
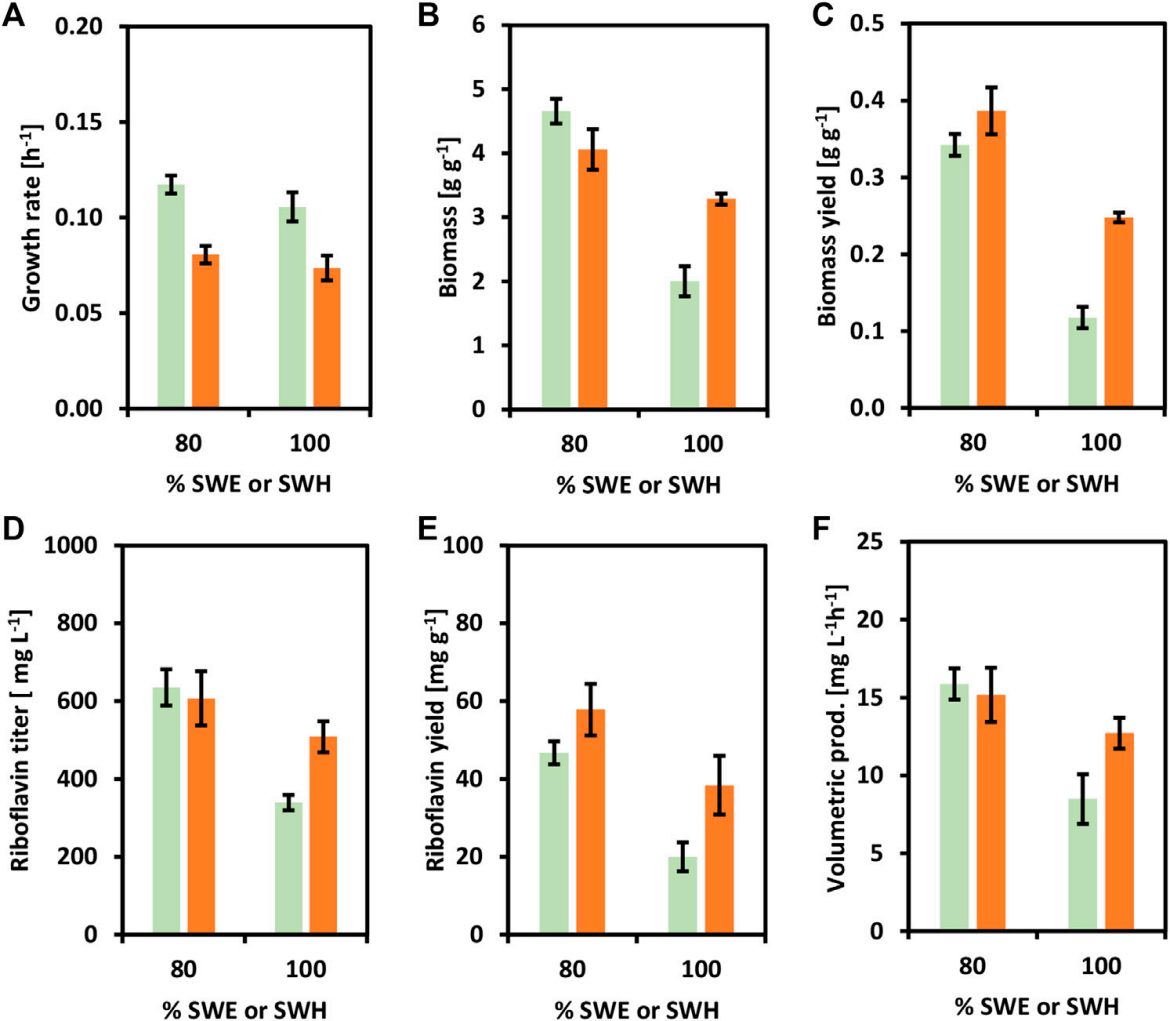
Growth and riboflavin production from brown seaweed-based substrates of the strain CgRibo4. SWE (green columns) or SWH (orange columns) were assayed in the concentrations of 80 and 100%. **(A)** Growth rates (h^−1^) of CgRibo4. **(B)** Final biomasses of formed (g L^−1^) CgRibo4. **(C)** Biomass yields (g g^−1^) of CgRibo4. **(D)** Final riboflavin titers (mg L^−1^) of CgRibo4. **(E)** Riboflavin yields (mg g^−1^) of CgRibo4. **(F)** Riboflavin volumetric productivities (mg L^−1^ h^−1^) of CgRibo4. Means and standard deviations of triplicates are shown.

The riboflavin producer CgRibo4 was also cultivated with SWE and SWH as carbon sources to prove the establishment of a newly added value chain from seaweed-based substrates. The concentrations of 80 and 100% of SWE or SWH were chosen as the most relevant conditions for the purpose of this study (Figure 7). As previously, when using 80% of SWE or SWH the medium contained all the components of the minimal medium as well. Similar to earlier results, the CgRibo4 strain grew faster on SWE, but the biomass yields were higher with SWH, especially when 100% SWH was used (Figures 7A–C). With regard to riboflavin, the best productivity values were achieved with 80% SWE. Under such conditions, a riboflavin titer of 635.0 ± 40.0 mg L^−1^ peaked after 40 h, indicating a volumetric productivity of 15.9 ± 1.0 mg L^−1^ h^−1^. However, the best riboflavin yield of 57.8 ± 6.6 mg g^−1^ was achieved from 80% SWH (Figures 7D–F). Mannitol and glucose were fully depleted by the end of the growth experiment.

Here, we have successfully established competitive riboflavin production in *C. glutamicum* strains from glucose and seaweed-based substrates. Production values up to this point are summarized in Table 6. Subsequently, the feasibility of using seaweed-based substrates as alternative microbial substrate was evaluated in bioreactor fermentations.

**TABLE 6:** Riboflavin production values from flask fermentations.

Strain	Carbon source	Titer	Yield	Vol. prod
		(mg L^−1^)	(mg g^−1^)	(mg L^−1^ h^−1^)
CgRibo2	0.25% glucose	165.7 ± 12.6	66.3 ± 5.0	4.1 ± 0.3
CgRibo2	0.5% glucose	381.0 ± 46.5	76.2 ± 9.3	9.5 ± 1.2
CgRibo2	1% glucose	613.1 ± 86.0	61.3 ± 8.6	12.3 ± 1.7
CgRibo2	2% glucose	746.9 ± 133.3	37.3 ± 6.7	14.9 ± 2.7
CgRibo2	4% glucose	944.6 ± 87.1	23.6 ± 2.2	18.9 ± 1.7
CgRibo4	1% mannitol	467.3 ± 45.7	46.7 ± 4.6	7.8 ± 0.8
CgRibo4	1% glucose	387.1 ± 32.7	38.7 ± 3.3	6.5 ± 0.5
CgRibo4	1% mannitol plus 1% glucose	607.3 ± 33.1	30.4 ± 3.3	10.1 ± 0.6
CgRibo4	80% SWE	635.0 ± 40.0	46.7 ± 2.9	15.9 ± 1.0
CgRibo4	100% SWE	339.5 ± 63.7	20.0 ± 3.7	8.5 ± 1.6
CgRibo4	80% SWH	606.8 ± 69.7	57.8 ± 6.6	15.2 ± 1.7
CgRibo4	100% SWH	508.5 ± 39.9	38.4 ± 7.5	12.7 ± 1.0

### Brown Seaweed-Based Fed-Batch Fermentations With *C. glutamicum* Overproducing Riboflavin

**FIGURE 8:**
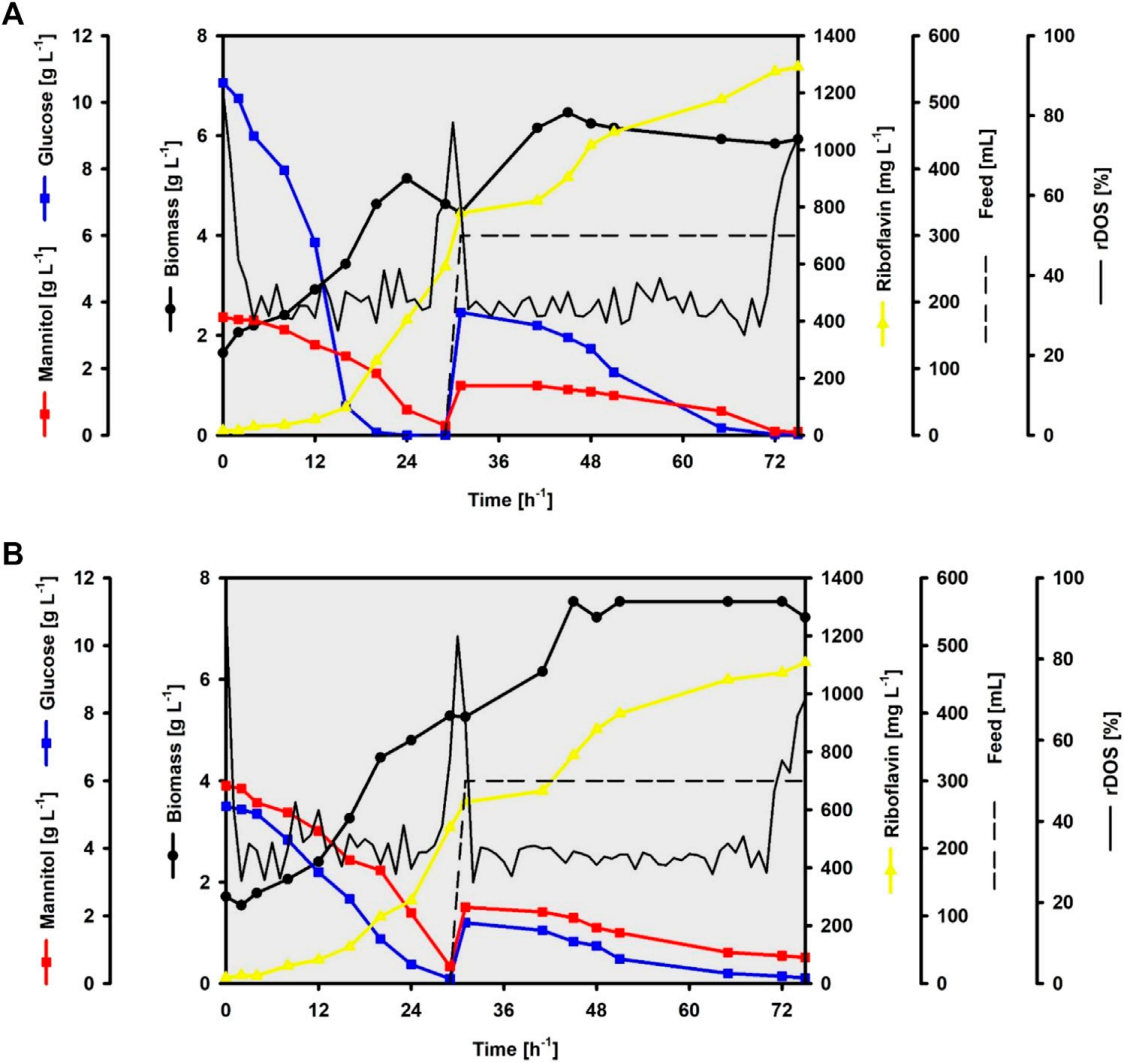
Fed-batch fermentations of the strain CgRibo4 using brown seaweed-based substrates. Glucose and mannitol concentrations are depicted with blue and red lines, respectively. Biomass concentrations are depicted with black circles. Riboflavin concentrations are depicted with yellow lines. The rDOS levels are depicted with black lines. Feed supplementations are depicted with dashed black lines. **(A)** SWE-based process. **(B)** SWH-based process.

To fully test the performance of CgRibo4, such strain was grown in lab-scale bioreactors following a fed-batch fermentation approach. SWE or SWH were used as carbon source in the concentrations of 80 and 100% for the batch and feed phases, respectively. According to HPLC measurements, the SWE-based fermentation media contained 3.5 g L^−1^ of mannitol and 10.6 g L^−1^ of glucose in the batch medium and 4.2 g L^−1^ of mannitol and 12.5 g L^−1^ of glucose in the feed medium. While the SWH-based fermentation contained 5.9 g L^−1^ of mannitol and 5.3 g L^−1^ of glucose in the batch medium and 6.9 g L^−1^ of mannitol and 6.2 g L^−1^ of glucose in the feed medium. A linear feeding rate of 2.5 ml min^−1^ was applied in both fermentations when the relative dissolved oxygen saturation (rDOS) value increased from 30% to 65% and above (after 30 h). The fermentation processes were considered completed when the rDOS value increased from 30% to 65% and above, during the feeding phase (after 75 h). By the end of the SWE-based fermentation, 0.1 g L^−1^ of mannitol was left, and 0.8 g L^−1^ of mannitol and 0.2 g L^−1^ of glucose were not used in the SWH-based fermentation. The riboflavin final titer, yield, and volumetric values of 1,291.2 mg L^−1^, 66.1 mg g^−1^, and 17.2 mg L^−1^ h^−1^, respectively, were achieved when using SWE (Figure 8A). On the other hand, 1,108.9 mg L^−1^, 75.8 mg g^−1^, and 14.8 mg L^−1^ h^−1^ values were achieved when using SWH (Figure 8B). While the highest riboflavin titer and volumetric productivity were achieved with SWE as carbon source, the highest riboflavin yield was reached when using SWH as carbon source. Differences were also observed in the new biomasses formed, since 4.8 g L^−1^ of biomass was produced from the SWE sugars while 5.5 g L^−1^ were produced from the SWH sugars, exhibiting biomass yields of 0.25 g g^−1^ and 0.38 g g^−1^, respectively. Interestingly, in both fermentations, the biomass reached steady state during the feeding phases, but riboflavin continued to be accumulated until the end of the processes.

Our bioreactor fermentations proved that the production of riboflavin can be based on seaweed substrates up to 100%, being also scalable to higher working volumes.

## Discussion

Nowadays, alternative feedstocks like wood-related residual biomass, crop residues, wastewater, or agri-food co-products are in the spotlight (Javourez et al., 2021). Biomass is a key sustainable feedstock for the transition toward circular and low fossil carbon economies. In particular, seaweed biomass is a promising candidate due to the lack of need for arable land, freshwater requirement for cultivation, and the presence of fermentable carbohydrates in their composition (Barbot et al., 2016). The total carbohydrate content among seaweed species varies from 24% to 65% in some brown seaweeds like *Laminaria digitata*, being comparable with the carbohydrate content of 65–75% in lignocellulosic biomass (Olsson et al., 2020). Mannitol, laminarin, alginate, and fucoidan in brown seaweeds are promising substrates for the utilization in biorefinery processes. In comparison, red seaweed varieties consist of different typical carbohydrates kinds including water-soluble sulfur-containing galactans like agar and carrageenan (Jiménez-Escrig and Sánchez-Muniz, 2000). Some seaweed species have been investigated as fermentation substrate previously. For instance, the red seaweed *Gracilaria* sp. was used as substrate for *Lactobacillus acidophilus* and *Lactobacillus plantarum* for lactic acid production (Monteiro et al., 2021). Regarding brown seaweeds, the species *Saccharina japonica* was fermented by *Monascus purpureus* to produce lovastatin (Suraiya et al., 2018). In another example, the *S. japonica* extracts containing mannitol and glucose were used to produce value-added chemicals like cadaverine and C30 terpenoids by *B. methanolicus* (Hakvåg et al., 2020). In this study, the brown seaweed *L. hyperborea* from the Norwegian coasts was shown to be a promising alternative microbial substrate for bioprocesses with the microbial workhorse *C. glutamicum*. Seaweed extract and hydrolysate contained mannitol and glucose from laminarin, which were targeted as fermentable sugars here. For most of the common biotechnological workhorses like *E. coli*, *B. subtilis*, or *C. glutamicum*, glucose is a native carbon source (Fujita, 2009; Lindner et al., 2011; Bren et al., 2016) and many efforts have been taken to fully understand and improve glucose utilization by these microbial workhorses (Fujita, 2009; Pérez-García et al., 2016; Alva et al., 2020). Regarding mannitol, some microorganisms have specific systems for the uptake and phosphorylation of mannitol, as it is the case for *E. coli*, *B. subtilis*, and *B. methanolicus*, which possess mannitol-specific PTSs (Figge et al., 1994; Heravi and Altenbuchner, 2014; López et al., 2019). The wild-type *C. glutamicum* grows on arabitol, but not on other sugar alcohols (Laslo et al., 2012). However, growth of *C. glutamicum* in the presence of arabitol allows subsequent growth on mannitol. Utilization of mannitol by *C. glutamicum* can be triggered by the overexpression of the arabitol/mannitol catabolic operon *mtlTD* (Peng et al., 2011).In addition, the deletion of the transcriptional repressor gene *mtlR* also led to the overexpression of *mtlTD* and, hence allowing mannitol utilization by *C. glutamicum* (Laslo et al., 2012). These approaches have been used to establish *C. glutamicum* mannitol-consuming strains (Laslo et al., 2012; Hoffmann et al., 2018). However, mannitol is not directly included in the central metabolism, but it is rather oxidized to fructose, which escapes the cells to be further assimilated and phosphorylated via the fructose-specific PTS (Laslo et al., 2012). In this study, the mannitol-specific PTSs from *E. coli*, *B. subtilis*, and *B. methanolicus* were tested in *C. glutamicum.* Mannitol-based growth was achieved by using the systems from *B. subtilis* and *B. methanolicus*. However, the system from *E. coli* could not enable mannitol-based growth in *C. glutamicum*. In a PTS system, the mannitol-specific components (EII) include three domains, the mannitol permease IIC (EIIC) component, the mannitol-specific phosphotransferase enzyme IIB (EIIB) component, and the mannitol-specific phosphotransferase enzyme IIA (EIIA) component (Clore and Venditti, 2013). While in *E. coli*, the EIICBA components are coded by *mtlA*, and in *B. subtilis* and *B. methanolicus*, the EIICB components are coded by *mtlA* and the EIIA component is coded by *mtlF* as independent protein (Heravi and Altenbuchner, 2014; López et al., 2019). This gene distribution may affect the interaction between the PTS components, and ultimately may disturb the final function of those proteins. The EIIA component is the direct phosphoryl group acceptor which is donated by the phosphocarrier HPr protein (Lindner et al., 2011). *C. glutamicum* ATCC 13032 has PTS genes encoding four different EII permeases, specific for glucose (PtsG), fructose (PtsF), sucrose (PtsS), and one unknown substrate (Moon et al., 2007). The *C. glutamicum* deletion mutant lacking the general PTS component HPr is unable to use the PTS systems for growth (Lindner et al., 2011). Therefore, it is important that HPr can interact with the EIIA component. Aligning protein sequences via BLASTP (Altschul et al., 1990), we observed that the EIIA components from *E. coli*, *B. subtilis*, and *B. methanolicus* present similarities in their protein sequences with PtsF from *C. glutamicum.* Query covers and identities for *E. coli*’s EIIA component were, 68 and 29% , 88 and 27% for *B. subtilis*’s EIIA component, and 72 and 36% for *B. methanolicus*’s EIIA component, respectively. According to these values, the EIIA component sequence from *B. subtilis* and *B. methanolicus* are closer to the sequence of PtsF from *C. glutamicum. B. methanolicus* is a thermotolerant microorganism with an optimum growth temperature of 50—53°C (Irla et al., 2014), hence the folding of MtlAF and MtlD may have been negatively affected when using 30°C during growth experiments with *C. glutamicum,* which supports the fact that the MtlAFD from *B. subtilis* performed best in *C. glutamicum*, since both are mesophilic microorganism (Droffner and Yamamoto, 1985; Eggeling et al., 2005). Nevertheless, *C. glut*amicum growth was shown from the seaweed-based substrates SWE and SWH. According to our results, SWE seems to be a less harmful substrate for the *C. glutamicum* cells in comparison to SWH. The presence of the furfural only in SWH might have contributed to growth inhibition even though *C. glutamicum* showed recalcitrance against growth inhibitors from some hydrolysates, such as methylfurfural and hydroxymethylfurfural (Gopinath et al., 2011; Mindt et al., 2019).

Industrial riboflavin production is traditionally carried out using the organisms *B. subtilis* and the filamentous fungus *Ashbya gossypii* (Schwechheimer et al., 2016; Averianova et al., 2020) reaching values up to 26.8 g L^−1^ of riboflavin *B. subtilis* strain KCCM 10445 (Lee et al., 2004). Some of the genetic modifications used for the development of riboflavin-producing strains include the overexpression of the riboflavin biosynthetic genes, increasing the metabolic flux towards the pentose phosphate pathway, or activation of the glycine and the purine pathways (Averianova et al., 2020). In *C. glutamicum*, a differential gene expression analysis revealed that the overexpression of sigma factor gene *sigH* upregulates the riboflavin biosynthesis genes *ribH*, *ribA*, and *ribC*, increasing the formation of riboflavin (Taniguchi and Wendisch, 2015). By overexpressing *sigH* and following a dynamic co-cultivation approach in bioreactors with a mixture of sugars, a riboflavin titer of 27 mg L^−1^ was achieved (Pérez-García et al., 2021). In this work, the riboflavin biosynthetic genes from *C. glutamicum* were constitutively overexpressed under the control of the synthetic *Psyn* promoter (Henke et al., 2021). The best production values were obtained with the strain CgRibo2 when using 4% of glucose as sole carbon source, peaking at 944.6 ± 87.1 mg L^−1^ of riboflavin in flask fermentations, showing a 35-fold higher titer than the previously reported by Pérez-García et al., in 2021. In a similar approach, a *B. subtilis* strain containing multiple copies of the endogenous riboflavin biosynthetic operon expressed constitutively from strong phage promoters produced 14 g L^−1^ after 48 h in fed-batch operating bioreactors (Perkins et al., 1999). However, the production values of the strain CgRibo4 decreased significantly compared to those of CgRibo2 using glucose. This may be explained by the metabolic burden of carrying three vectors in CgRibo4 instead of one vector in CgRibo2 (Wein et al., 2019). Yet, riboflavin production was also shown from seaweed-based substrates for the first time here. The best riboflavin titer of 1,291.2 mg L^−1^ was achieved after 75 h using SWE as sole carbon source in a fed-batch fermentation. The fed-batch method is a common approach that has been applied to riboflavin industrial strains (Averianova et al., 2020). For instance, production of riboflavin with *A. gossypii* is carried out in fed-batch fermentations with aerobic conditions during 6–8 days (Stahmann et al., 2000). In another example, the process of fermentation for riboflavin production with the *B. subtilis* strain RB 50 is carried out on carbon-limited fed-batch cultivations during 48–56 h (Bretzel et al., 1999). Nevertheless, the feasibility of the newly generated riboflavin producer was successfully tested in seaweed-based fed-batch fermentations proving to be a promising process. In this regard, *de novo* production via microbial fermentations offers the flexibility for using broad natural substrates or non-natural carbon sources prior microbial engineering (Wendisch et al., 2016). Moreover, alternative renewable feedstocks or carbon containing waste streams have the potential to become more attractive within these processes (Gopinath et al., 2011; Buschke et al., 2013; Meiswinkel et al., 2013; Mindt et al., 2019).

In conclusion, we have established *C. glutamicum* as a promising riboflavin production host that utilizes brown seaweed-derived carbohydrates as feedstock. Heterologous expression of the mannitol-specific PTS together with mannitol-1-phosphate 5-dehydrogenase from *B. subtilis* enabled efficient utilization of mannitol by *C. glutamicum.* Combined with the constitutive overexpression of the endogenous riboflavin biosynthetic operon from *C. glutamicum,* we generated the *C. glutamicum* strain with the highest riboflavin production values regarding titer, yield, and productivity from glucose. Moreover, we have shown the production of riboflavin from seaweed-based substrates in flask and bioreactor fermentations.

## Data Availability

The original contributions presented in the study are included in the article/Supplementary Material, further inquiries can be directed to the corresponding author.

## References

[B1] Abe S. Takayama K.-I. Kinoshita S. (1967). Taxonomical Studies on Glutamic Acid-Producing Bacteria. J. Gen. Appl. Microbiol. 13, 279–301. 10.2323/jgam.13.279

[B2] Alderkamp A.-C. Van Rijssel M. Bolhuis H. (2007). Characterization of marine Bacteria and the Activity of Their Enzyme Systems Involved in Degradation of the Algal Storage Glucan Laminarin. FEMS Microbiol. Ecol. 59, 108–117. 10.1111/j.1574-6941.2006.00219.x 17233748

[B3] Altschul S. F. Gish W. Miller W. Myers E. W. Lipman D. J. (1990). Basic Local Alignment Search Tool. J. Mol. Biol. 215, 403–410. 10.1016/S0022-2836(05)80360-2 2231712

[B4] Alva A. Sabido-Ramos A. Escalante A. Bolívar F. (2020). New Insights into Transport Capability of Sugars and its Impact on Growth from Novel Mutants of *Escherichia coli* . Appl. Microbiol. Biotechnol. 104, 1463–1479. 10.1007/s00253-019-10335-x 31900563

[B5] Averianova L. A. Balabanova L. A. Son O. M. Podvolotskaya A. B. Tekutyeva L. A. (2020). Production of Vitamin B2 (Riboflavin) by Microorganisms: An Overview. Front. Bioeng. Biotechnol. 8, 1172. 10.3389/fbioe.2020.570828 PMC7693651 33304888

[B6] Barbot Y. Al-Ghaili H. Benz R. (2016). A Review on the Valorization of Macroalgal Wastes for Biomethane Production. Mar. Drugs 14, 120. 10.3390/md14060120 PMC4926079 27338422

[B7] Becker J. Rohles C. M. Wittmann C. (2018). Metabolically Engineered *Corynebacterium Glutamicum* for Bio-Based Production of Chemicals, Fuels, Materials, and Healthcare Products. Metab. Eng. 50, 122–141. 10.1016/j.ymben.2018.07.008 30031852

[B8] Becker S. Tebben J. Coffinet S. Wiltshire K. Iversen M. H. Harder T. (2020). Laminarin Is a Major Molecule in the marine Carbon Cycle. Proc. Natl. Acad. Sci. U.S.A. 117, 6599–6607. 10.1073/pnas.1917001117 32170018 PMC7104365

[B9] Bren A. Park J. O. Towbin B. D. Dekel E. Rabinowitz J. D. Alon U. (2016). Glucose Becomes One of the Worst Carbon Sources for E.Coli on Poor Nitrogen Sources Due to Suboptimal Levels of cAMP. Sci. Rep. 6, 24834. 10.1038/srep24834 27109914 PMC4843011

[B10] Bretzel W. Schurter W. Ludwig B. Kupfer E. Doswald S. Pfister M. (1999). Commercial Riboflavin Production by Recombinant Bacillus Subtilis : Down-Stream Processing and Comparison of the Composition of Riboflavin Produced by Fermentation or Chemical Synthesis. J. Ind. Microbiol. Biotechnol. 22, 19–26. 10.1038/sj.jim.2900604

[B11] Buschke N. Schäfer R. Becker J. Wittmann C. (2013). Metabolic Engineering of Industrial Platform Microorganisms for Biorefinery Applications - Optimization of Substrate Spectrum and Process Robustness by Rational and Evolutive Strategies. Bioresour. Tech. 135, 544–554. 10.1016/j.biortech.2012.11.047 23260271

[B12] Clore G. M. Venditti V. (2013). Structure, Dynamics and Biophysics of the Cytoplasmic Protein-Protein Complexes of the Bacterial Phosphoenolpyruvate: Sugar Phosphotransferase System. Trends Biochem. Sci. 38, 515–530. 10.1016/j.tibs.2013.08.00310.1016/j.tibs.2013.08.003 24055245 PMC3831880

[B13] Droffner M. L. Yamamoto N. (1985). Isolation of Thermophilic Mutants of *Bacillus Subtilis* and *Bacillus Pumilus* and Transformation of the Thermophilic Trait to Mesophilic Strains. Microbiology 131, 2789–2794. 10.1099/00221287-131-10-2789 3934332

[B14] Eggeling L. Bott M. Bott M. (2005). Handbook of Corynebacterium Glutamicum. CRC Press. 10.1201/9781420039696

[B15] Figge R. M. Ramseier T. M. Saier M. H. (1994). The Mannitol Repressor (MtlR) of *Escherichia coli* . J. Bacteriol. 176, 840–847. 10.1128/jb.176.3.840-847.1994 8300537 PMC205122

[B16] Fujita Y. (2009). Carbon Catabolite Control of the Metabolic Network inBacillus Subtilis. Biosci. Biotechnol. Biochem. 73, 245–259. 10.1271/bbb.80479 19202299

[B17] Gibson D. G. Young L. Chuang R.-Y. Venter J. C. Hutchison C. A. Smith H. O. (2009). Enzymatic Assembly of DNA Molecules up to Several Hundred Kilobases. Nat. Methods 6, 343–345. 10.1038/nmeth.1318 19363495

[B18] Gopinath V. Meiswinkel T. M. Wendisch V. F. Nampoothiri K. M. (2011). Amino Acid Production from rice Straw and Wheat Bran Hydrolysates by Recombinant Pentose-Utilizing *Corynebacterium Glutamicum* . Appl. Microbiol. Biotechnol. 92, 985–996. 10.1007/s00253-011-3478-x 21796382

[B19] Görke B. Stülke J. (2008). Carbon Catabolite Repression in Bacteria: many Ways to Make the Most Out of Nutrients. Nat. Rev. Microbiol. 6, 613–624. 10.1038/nrmicro1932 18628769

[B20] Green M. R. Sambrook J. (2012). Molecular Cloning: A Laboratory Manual. Cold Spring Harbor Laboratory Press.

[B21] Hakvåg S. Nærdal I. Heggeset T. M. B. Kristiansen K. A. Aasen I. M. Brautaset T. (2020). Production of Value-Added Chemicals by Bacillus Methanolicus Strains Cultivated on Mannitol and Extracts of Seaweed Saccharina Latissima at 50°C. Front. Microbiol. 11, 680. 10.3389/fmicb.2020.00680 32328058 PMC7161427

[B22] Hanahan D. (1983). Studies on Transformation of *Escherichia coli* with Plasmids. J. Mol. Biol. 166, 557–580. 10.1016/S0022-2836(83)80284-8 6345791

[B23] Henke N. A. Krahn I. Wendisch V. F. (2021). Improved Plasmid-Based Inducible and Constitutive Gene Expression in *Corynebacterium Glutamicum* . Microorganisms 9, 204. 10.3390/microorganisms9010204 33478126 PMC7835838

[B24] Heravi K. M. Altenbuchner J. (2014). Regulation of the *Bacillus Subtilis* Mannitol Utilization Genes: Promoter Structure and Transcriptional Activation by the Wild-type Regulator (MtlR) and its Mutants. Microbiology (Reading) 160, 91–101. 10.1099/mic.0.071233-0 24196428

[B25] Hoffmann S. L. Jungmann L. Schiefelbein S. Peyriga L. Cahoreau E. Portais J.-C. (2018). Lysine Production from the Sugar Alcohol Mannitol: Design of the Cell Factory *Corynebacterium Glutamicum* SEA-3 through Integrated Analysis and Engineering of Metabolic Pathway Fluxes. Metab. Eng. 47, 475–487. 10.1016/j.ymben.2018.04.019 29709649

[B26] Irla M. Neshat A. Winkler A. Albersmeier A. Heggeset T. M. B. Brautaset T. (2014). Complete Genome Sequence of *Bacillus Methanolicus* MGA3, a Thermotolerant Amino Acid Producing Methylotroph. J. Biotechnol. 188, 110–111. 10.1016/j.jbiotec.2014.08.013 25152427

[B27] Javourez U. O’Donohue M. Hamelin L. (2021). Waste-to-nutrition: a Review of Current and Emerging Conversion Pathways. Biotechnol. Adv. 53, 107857. 10.1016/j.biotechadv.2021.107857 34699952

[B28] Jiménez-Escrig A. Sánchez-Muniz F. J. (2000). Dietary Fibre from Edible Seaweeds: Chemical Structure, Physicochemical Properties and Effects on Cholesterol Metabolism. Nutr. Res. 20, 585–598. 10.1016/S0271-5317(00)00149-4

[B29] Laslo T. von Zaluskowski P. Gabris C. Lodd E. Ruckert C. Dangel P. (2012). Arabitol Metabolism of *Corynebacterium Glutamicum* and its Regulation by AtlR. J. Bacteriol. 194, 941–955. 10.1128/JB.06064-11 22178972 PMC3294798

[B30] Lee K. H. Choi H. Han J. K. Park Y. H. Park J. H. (2004). Microorganisms and Process for the Production of Riboflavin by Fermentation, 809–1401. Available at: https://patents.google.com/patent/EP1426450A1/en (Accessed December 5, 2021).

[B31] Lindner S. N. Seibold G. M. Henrich A. Krämer R. Wendisch V. F. (2011). Phosphotransferase System-independent Glucose Utilization in *Corynebacterium Glutamicum* by Inositol Permeases and Glucokinases. Appl. Environ. Microbiol. 77, 3571–3581. 10.1128/AEM.02713-10 21478323 PMC3127631

[B32] Liu S. Hu W. Wang Z. Chen T. (2020). Production of Riboflavin and Related Cofactors by Biotechnological Processes. Microb. Cel Fact. 19, 31. 10.1186/s12934-020-01302-7 PMC7017516 32054466

[B33] López M. G. Irla M. Brito L. F. Wendisch V. F. (2019). Characterization of D-Arabitol as Newly Discovered Carbon Source of *Bacillus Methanolicus* . Front. Microbiol. 10. 10.3389/fmicb.2019.01725 PMC6685057 31417519

[B34] Meiswinkel T. M. Rittmann D. Lindner S. N. Wendisch V. F. (2013). Crude Glycerol-Based Production of Amino Acids and Putrescine by *Corynebacterium Glutamicum* . Bioresour. Tech. 145, 254–258. 10.1016/j.biortech.2013.02.053 23562176

[B35] Mindt M. Hannibal S. Heuser M. Risse J. M. Sasikumar K. Nampoothiri K. M. (2019). Fermentative Production of N-Alkylated Glycine Derivatives by Recombinant *Corynebacterium Glutamicum* Using a Mutant of Imine Reductase DpkA from Pseudomonas Putida. Front. Bioeng. Biotechnol. 7, 232. 10.3389/fbioe.2019.00232 31616665 PMC6775277

[B36] Monteiro P. Lomartire S. Cotas J. Pacheco D. Marques J. C. Pereira L. (2021). Seaweeds as a Fermentation Substrate: A Challenge for the Food Processing Industry. Processes 9, 1953. 10.3390/pr9111953

[B37] Moon M.-W. Park S.-Y. Choi S.-K. Lee J.-K. (2007). The Phosphotransferase System of *Corynebacterium Glutamicum*: Features of Sugar Transport and Carbon Regulation. J. Mol. Microbiol. Biotechnol. 12, 43–50. 10.1159/000096458 17183210

[B38] Olsson J. Toth G. B. Albers E. (2020). Biochemical Composition of Red, green and Brown Seaweeds on the Swedish West Coast. J. Appl. Phycol 32, 3305–3317. 10.1007/s10811-020-02145-w

[B39] Peng X. Okai N. Vertès A. A. Inatomi K.-i. Inui M. Yukawa H. (2011). Characterization of the Mannitol Catabolic Operon of *Corynebacterium Glutamicum* . Appl. Microbiol. Biotechnol. 91, 1375–1387. 10.1007/s00253-011-3352-x 21655984

[B40] Pérez-García F. Burgardt A. Kallman D. R. Wendisch V. F. Bar N. (2021). Dynamic Co-cultivation Process of *Corynebacterium Glutamicum* Strains for the Fermentative Production of Riboflavin. Fermentation 7, 11. 10.3390/fermentation7010011

[B41] Pérez-García F. Peters-Wendisch P. Wendisch V. F. (2016). Engineering *Corynebacterium Glutamicum* for Fast Production of L-Lysine and L-Pipecolic Acid. Appl. Microbiol. Biotechnol. 100, 8075–8090. 10.1007/s00253-016-7682-6 27345060

[B42] Pérez-García F. Wendisch V. F. (2018). Transport and Metabolic Engineering of the Cell Factory *Corynebacterium Glutamicum* . FEMS Microbiol. Lett. 365. 10.1093/femsle/fny166 29982619

[B43] Perkins J. B. Sloma A. Hermann T. Theriault K. Zachgo E. Erdenberger T. (1999). Genetic Engineering of *Bacillus Subtilis* for the Commercial Production of Riboflavin. J. Ind. Microbiol. Biotechnol. 22, 8–18. 10.1038/sj.jim.2900587

[B44] Petteys B. J. Frank E. L. (2011). Rapid Determination of Vitamin B2 (Riboflavin) in Plasma by HPLC. Clinica Chim. Acta 412, 38–43. 10.1016/j.cca.2010.08.037 20816949

[B45] Pinto J. T. Zempleni J. (2016). Riboflavin. Adv. Nutr. 7, 973–975. 10.3945/an.116.012716 27633112 PMC5015041

[B46] Revuelta J. L. Ledesma-Amaro R. Lozano-Martinez P. Díaz-Fernández D. Buey R. M. Jiménez A. (2017). Bioproduction of Riboflavin: a Bright Yellow History. J. Ind. Microbiol. Biotechnol. 44, 659–665. 10.1007/s10295-016-1842-7 27696023

[B47] Schwechheimer S. K. Park E. Y. Revuelta J. L. Becker J. Wittmann C. (2016). Biotechnology of Riboflavin. Appl. Microbiol. Biotechnol. 100, 2107–2119. 10.1007/s00253-015-7256-z 26758294

[B48] Song S. H. Vieille C. (2009). Recent Advances in the Biological Production of Mannitol. Appl. Microbiol. Biotechnol. 84, 55–62. 10.1007/s00253-009-2086-5 19578847

[B49] Stahmann K.-P. Revuelta J. L. Seulberger H. (2000). Three Biotechnical Processes Using *Ashbya Gossypii, Candida Famata*, or *Bacillus Subtilis* Compete with Chemical Riboflavin Production. Appl. Microbiol. Biotechnol. 53, 509–516. 10.1007/s002530051649 10855708

[B50] Stansen C. Uy D. Delaunay S. Eggeling L. Goergen J.-L. Wendisch V. F. (2005). Characterization of a *Corynebacterium Glutamicum* Lactate Utilization Operon Induced during Temperature-Triggered Glutamate Production. Appl. Environ. Microbiol. 71, 5920–5928. 10.1128/AEM.71.10.5920-5928.2005 16204505 PMC1265975

[B51] Suraiya S. Kim J.-H. Tak J. Y. Siddique M. P. Young C. J. Kim J. K. (2018). Influences of Fermentation Parameters on Lovastatin Production by *Monascus purpureus* Using *Saccharina Japonica* as Solid Fermented Substrate. LWT 92, 1–9. 10.1016/j.lwt.2018.02.013

[B52] Taniguchi H. Wendisch V. F. (2015). Exploring the Role of Sigma Factor Gene Expression on Production by *Corynebacterium Glutamicum*: Sigma Factor H and FMN as Example. Front. Microbiol. 6, 740. 10.3389/fmicb.2015.00740 26257719 PMC4510997

[B53] Wein T. Hülter N. F. Mizrahi I. Dagan T. (2019). Emergence of Plasmid Stability under Non-selective Conditions Maintains Antibiotic Resistance. Nat. Commun. 10, 2595. 10.1038/s41467-019-10600-7 31197163 PMC6565834

[B54] Wendisch V. F. Brito L. F. Gil Lopez M. Hennig G. Pfeifenschneider J. Sgobba E. (2016). The Flexible Feedstock Concept in Industrial Biotechnology: Metabolic Engineering of *Escherichia coli, Corynebacterium Glutamicum, Pseudomonas, Bacillus* and Yeast Strains for Access to Alternative Carbon Sources. J. Biotechnol. 234, 139–157. 10.1016/j.jbiotec.2016.07.022 27491712

[B55] Zhang B. Jiang Y. Li Z. Wang F. Wu X.-Y. (2020). Recent Progress on Chemical Production from Non-food Renewable Feedstocks Using *Corynebacterium Glutamicum* . Front. Bioeng. Biotechnol. 8, 606047. 10.3389/fbioe.2020.606047 33392171 PMC7775722

